# Supplementary material for: Phosphite as an engineered niche for Pseudomonas veronii in a synthetic soil bacterial community
Source: mSystems. 2025 Aug 15;10(9):e00061-25. doi: 10.1128/msystems.00061-25 (PMC12455932; doi:10.1128/msystems.00061-25)
Supplement: Supplemental Information — Tables S1–S3, Figures S1–S12, and supplemental methods. [file msystems.00061-25-s0001.docx]

Supplemental Information for

**Phosphite as an Engineered Niche for *Pseudomonas veronii* in a Synthetic Soil Bacterial Community**

Clara Bailey,^a^ Philip Gwyther,^b^ Senka Čaušević,^b^ Brandon L. Greene,^a,c^ and Jan Roelof van der Meer^b^

^a^ Department of Chemistry and Biochemistry, University of California, Santa Barbara, Santa Barbara, California, United States

^b^ Department of Fundamental Microbiology, University of Lausanne, Lausanne, Switzerland

^c^ Biomolecular Science and Engineering Program, University of California, Santa Barbara, Santa Barbara, California, United States

Corresponding authors: Clara Bailey, [clarabailey@ucsb.edu](mailto:clarabailey@ucsb.edu), Jan Roelof van der Meer, JanRoelof.VanDerMeer@unil.ch and Brandon L. Greene, greene@chem.ucsb.edu

Table of Contents

**Table 1.** SynCom members S3

**Table 2.** Primers and DNA sequences S4

**Figure 1.** *P. veronii ptxD*^+^ gene insertion and functionality S5

**Figure 2.** SynCom grow vs stable alpha diversity S6

**Figure 3.** NMDS plot of SynCom +/- *P. v* in the presence and absence of Phi S7

**Figure 4.** Toluene tolerance of SynCom S8

**Figure 5.** SynCom stacked relative abundances with toluene exposure S9

**Figure 6.** SynCom alpha diversity +/- toluene S10

**Figure 7.** NMDS plot of SynCom with and without toluene S11

**Figure 8.** SynCom stacked absolute abundances with toluene exposure, +/- *P. v* S12

**Figure 9.** NMDS plot of SynCom and *P. v* with toluene, +/- Phi S13

**Figure 10.** SynCom stacked relative abundances in SE S14

**Figure 11.** Cell densities in SE S15

**Figure 12.** SynCom strains on R2A agar S16

**Table 3.** BLAST Searches for *ptxD* in SynCom Members S17

**Supplemental Methods** S18–S23

Construction of *P. veronii ptxD*^+^ S18–S20

Community 16S rRNA Gene Amplicon Sequencing S20

Toluene Quantification by GC-MS S21

Statistical Analyses S21–S22

**References** S22–S23

Supplemental Table 1. SynCom members.

| Genus | Class | Phyla |
| --- | --- | --- |
| *Microbacterium* | Actinobacteria | Actinobacteria |
| *Mucilaginibacter* | Bacteroidia | Bacteroidetes |
| *Curtobacterium* | Actinobacteria | Actinobacteria |
| *Variovorax* | Gammaproteobacteria | Proteobacteria |
| *Flavobacterium* | Bacteroidia | Bacteroidetes |
| *Cellulomonas* | Actinobacteria | Actinobacteria |
| *Tardiphaga* | Alphaproteobacteria | Proteobacteria |
| *Devosia* | Alphaproteobacteria | Proteobacteria |
| *Mesorhizobium* | Alphaproteobacteria | Proteobacteria |
| *Burkholderia* | Betaproteobacteria | Proteobacteria |
| *Pseudomonas (1)* | Gammaproteobacteria | Proteobacteria |
| *Luteibacter* | Gammaproteobacteria | Proteobacteria |
| *Chitinophaga* | Bacteroidia | Bacteroidetes |
| *Lysobacter* | Gammaproteobacteria | Proteobacteria |
| *Pseudomonas (2)* | Gammaproteobacteria | Proteobacteria |
| *Rhodococcus* | Actinobacteria | Actinobacteria |
| *Caulobacter* | Alphaproteobacteria | Proteobacteria |
| *Cohnella* | Bacilli | Firmicutes |
| *Rahnella* | Gammaproteobacteria | Proteobacteria |
| *Phenylobacterium* | Alphaproteobacteria | Proteobacteria |
| *Bradyrhizobium* | Alphaproteobacteria | Proteobacteria |

Supplemental Table 2. Primers and DNA sequences used in this study.

| Name | Sequence | Purpose |
| --- | --- | --- |
| P(PstS) | 5′-GGTGCGGCGGCCTGGGCTTTGCCAGAGGCCATCACTCCCCTTTCTGATCGTCTGTCAGGAGCGGGCAAGGCTCAGACTTTGCCCCCTCAGCCTCTCCCCCAAAAAACAAAGGGCGAAACAAGGCTAGACCACCTAATGCACAACGGCCAGGCCCTAGCGCCTGGCGCGGTTGGCGCGACCCGCCATTCAACTTTCATAAAAGCTTAACGAAAGTGCTGCAGAGTTCGGGAGCCCGTTATCACAATGAGTTCCTTCGATGAAAAATTTGATGAAGTCTGCTGCACTCGCCGTTGCGGTTTCTCTTTGTGCAAGTTCCATGGGGAGGACTACTG-3′ | Promoter region upstream of *ptxD* in pUC18-Tn*7*-Gm-Amp-*ptxD*, originally the promoter region for *P. veronii* 1YdBTEX2 PstS gene. |
| *ptxD* | 5’- ATGCTGCCGAAACTCGTTATAACTCACCGAGTACACGATGAGATCCTGCAACTGCTGGCGCCACATTGCGAGCTGATGACCAACCAGACCGACAGCACGCTGACGCGCGAGGAAATTCTGCGCCGCTGTCGCGATGCTCAGGCGATGATGGCGTTCATGCCCGATCGGGTCGATGCAGACTTTCTTCAAGCCTGCCCTGAGCTGCGTGTAGTCGGCTGCGCGCTCAAGGGCTTCGACAATTTCGATGTGGACGCCTGTACTGCCCGCGGGGTCTGGCTGACCTTCGTGCCTGATCTGTTGACGGTCCCGACTGCCGAGCTGGCGATCGGACTGGCGGTGGGGCTGGGGCGGCATCTGCGGGCAGCAGATGCGTTCGTCCGCTCTGGCGAGTTCCAGGGCTGGCAACCACAGTTCTACGGCACGGGGCTGGATAACGCTACGGTCGGCATCCTTGGCATGGGCGCCATCGGACTGGCGATGGCTGATCGCTTGCAGGGATGGGGCGCGACCCTGCAGTACCACGAGGCGAAGGCTCTGGATACACAAACCGAGCAACGGCTCGGCCTGCGCCAGGTGGCGTGCAGCGAACTCTTCGCCAGCTCGGACTTCATCCTGCTGGCGCTTCCCTTGAATGCCGATACCCAGCATCTGGTCAACGCCGAGCTGCTTGCCCTCGTACGGCCGGGCGCTCTGCTTGTAAACCCCTGTCGTGGTTCGGTAGTGGATGAAGCCGCCGTGCTCGCGGCGCTTGAGCGAGGCCAGCTCGGCGGGTATGCGGCGGATGTATTCGAAATGGAAGACTGGGCTCGCGCGGACCGGCCGCGGCTGATCGATCCTGCGCTGCTCGCGCATCCGAATACGCTGTTCACTCCGCACATAGGGTCGGCAGTGCGCGCGGTGCGCCTGGAGATTGAACGTTGTGCAGCGCAGAACATCATCCAGGTATTGGCAGGTGCGCGCCCAATCAACGCTGCGAACCGTCTGCCCAAGGCCGAGCCTGCCGCATGTTGA | Sequence coding for PTDH. |
| *ptxD* Fwd | 5′-ATTCGATCATGCATGAGCTCACT-3′ | Insertion of P(PstS)-PTDH fragment into pUC18-Tn7-Gm-Amp vector. |
| *ptxD* Rev | 5′-GAGGTACCGGGCCCAA-3′ |  |
| V3/V4 Fwd | 5′-TCGTCGGCAGCGTCAGATGTGTATAAGAGACAGCCTACGGGNGGCWGCAG-3′ | Amplification of 16S V3/V4 region for 16S rRNA gene amplicon sequencing. |
| V3/V4 Rev | 5′-GTCTCGTGGGCTCGGAGATGTGTATAAGAGACAGGACTACHVGGGTATCTAATCC-3′ |  |


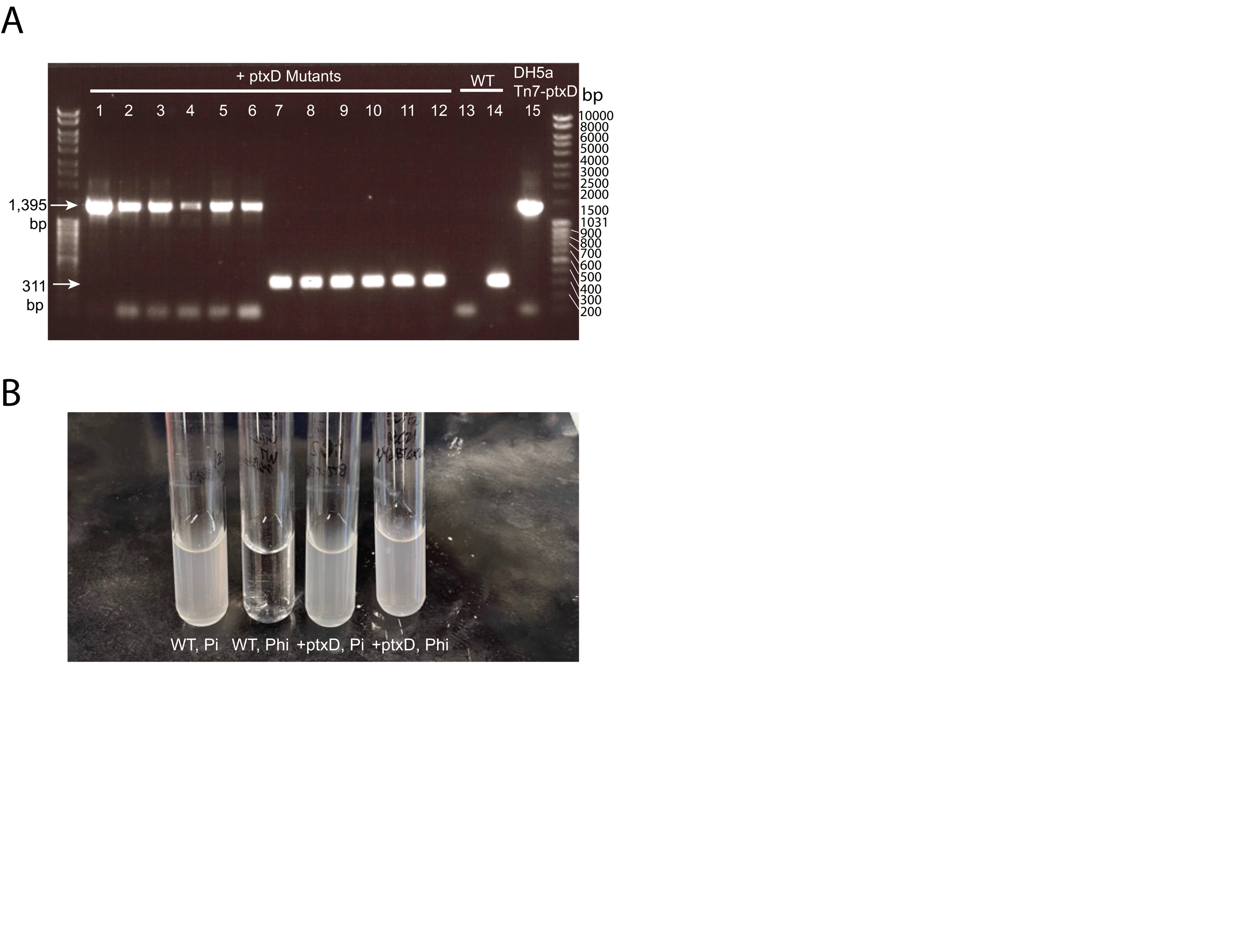


**Supplemental Figure 1**. Confirming the insertion and functionality of the *ptxD* gene in *P. veronii* 1YdBTEX2. **A** Agarose gel electrophoretic mobility describing the isolated mutant strain (lanes 1–12). The *ptxD* insertion is shown by the amplification of the 1,395 bp sequence by colony PCR (lanes 1–6). The amplification of the same sequence from a colony of DH5a *E. coli* containing the pUC18-Tn*7*-Gm-Amp-PTDH plasmid is shown as a positive control (lane 15), and from wild type (WT) *P. veronii* as a negative control (lane 13). A 311 bp sequence on chromosome 2 of *P. veronii* was amplified in the mutant strain by colony PCR to confirm the species identity (lanes 7–12). WT *P. veronii* was amplified using the same sequence primers as a positive control (lane 14). **B** Growth of WT *P. veronii* and the *P. veronii ptxD^+^* mutant on 21C media containing either phosphate (Pi) or phosphite (Phi) as the sole phosphorus source.


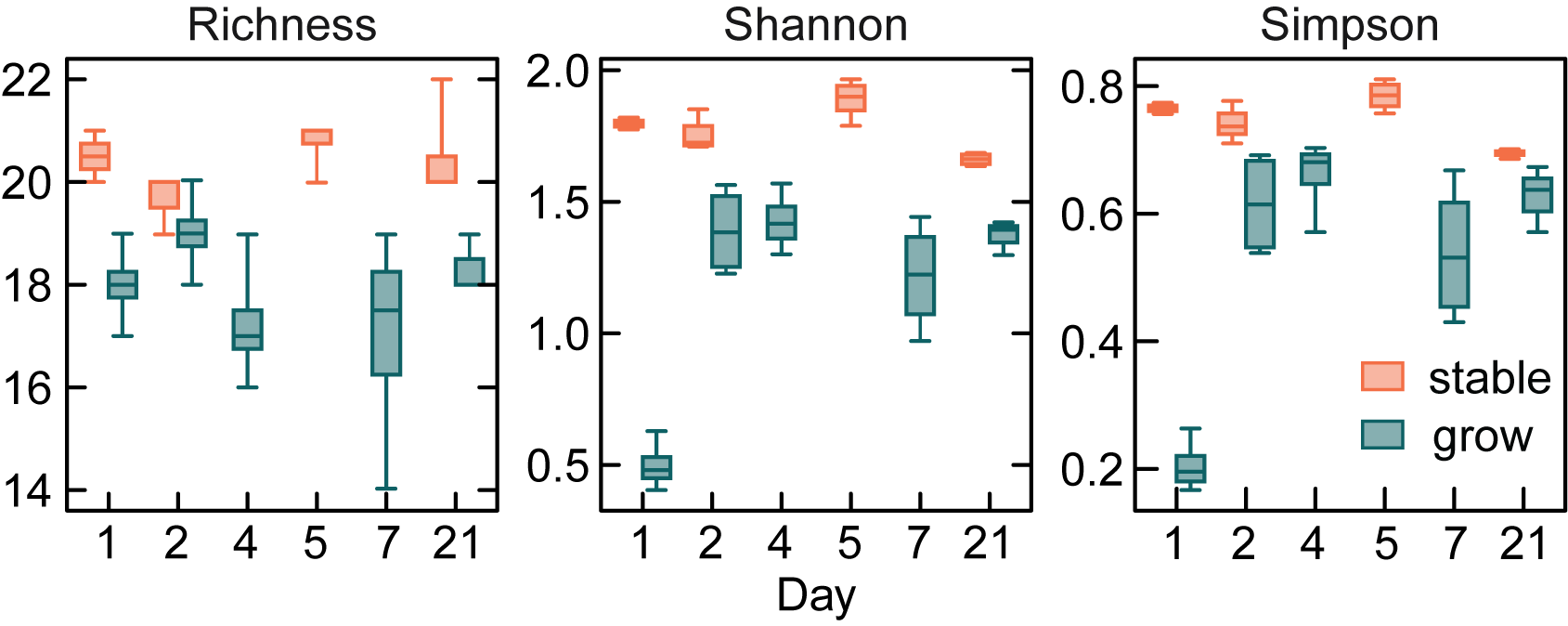


**Supplemental Figure 2.** Bar plots showing alpha diversity of SynCom in the grow (dark aqua) and stable (orange) phase according to three metrics: richness (p = 3.425 $\times$ 10^–7^), Shannon index (p = 1.716 $\times$ 10^–6^), and Simpson index (p = 6.984 $\times$ 10^–5^).


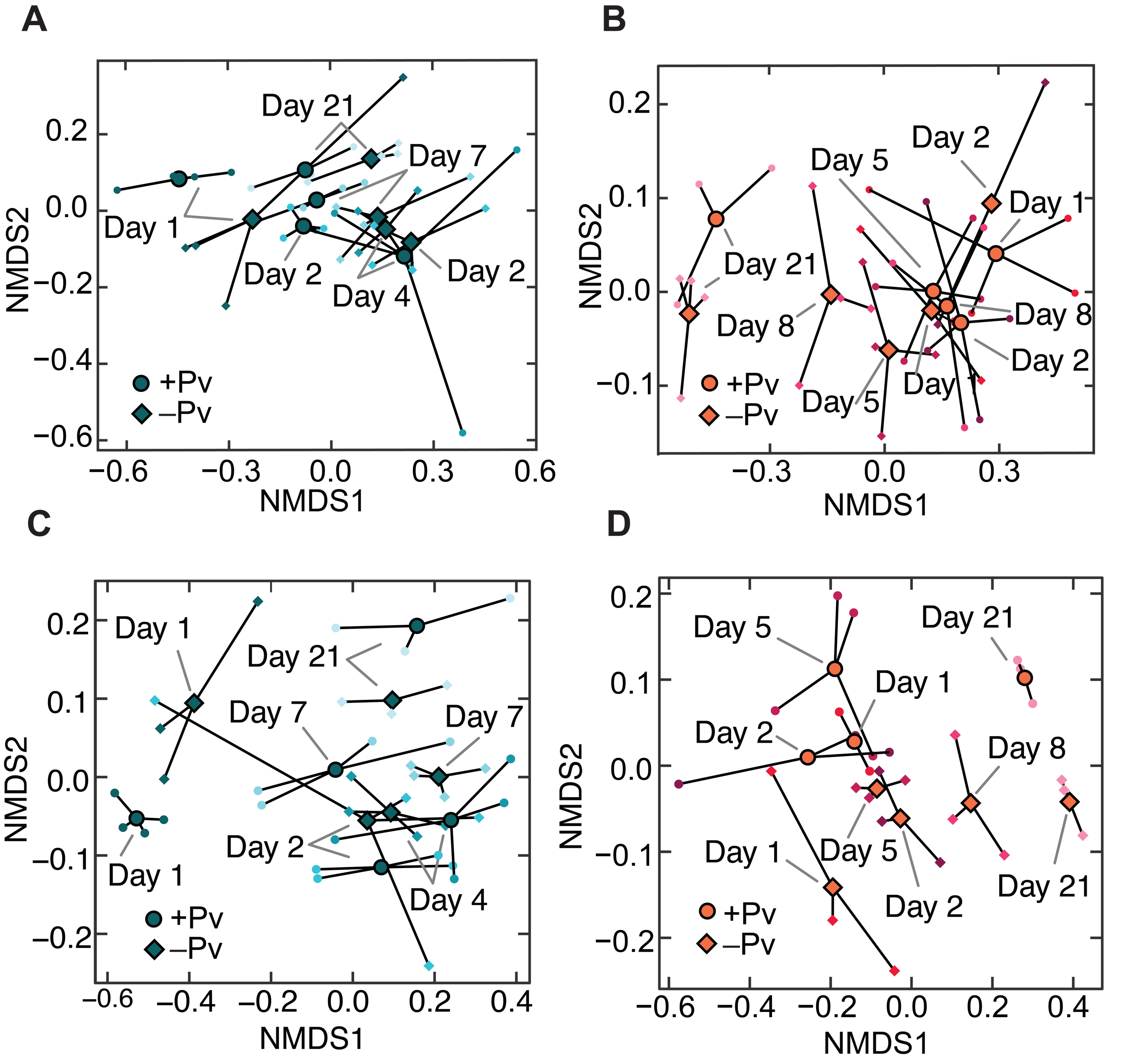


**Supplemental Figure 3.** Non-metric multidimensional scaling analysis of the SynCom species relative abundance data with and without *P. veronii* *ptxD^+^* inoculation in the presence of Phi during the grow (**A**, p = 0.0040 , two-way ANOVA) and stable (**B**, p = 0.0020, two-way ANOVA) phase of development over time using Bray-Curtis distance values (stress = 0.089 and 0.076 for grow and stable phase, respectively). Similar analysis is shown in the absence of Phi for grow (**C**, p = 0.185, two-way ANOVA) and stable (**D**, p = 0.0030, two-way ANOVA) phase SynCom (stress = 0.073 and 0.040 for grow and stable phase, respectively). Daily centroids are shown as outlined circles and diamonds and replicates are shown as smaller circles and diamonds, colored according to timepoint and community phase.


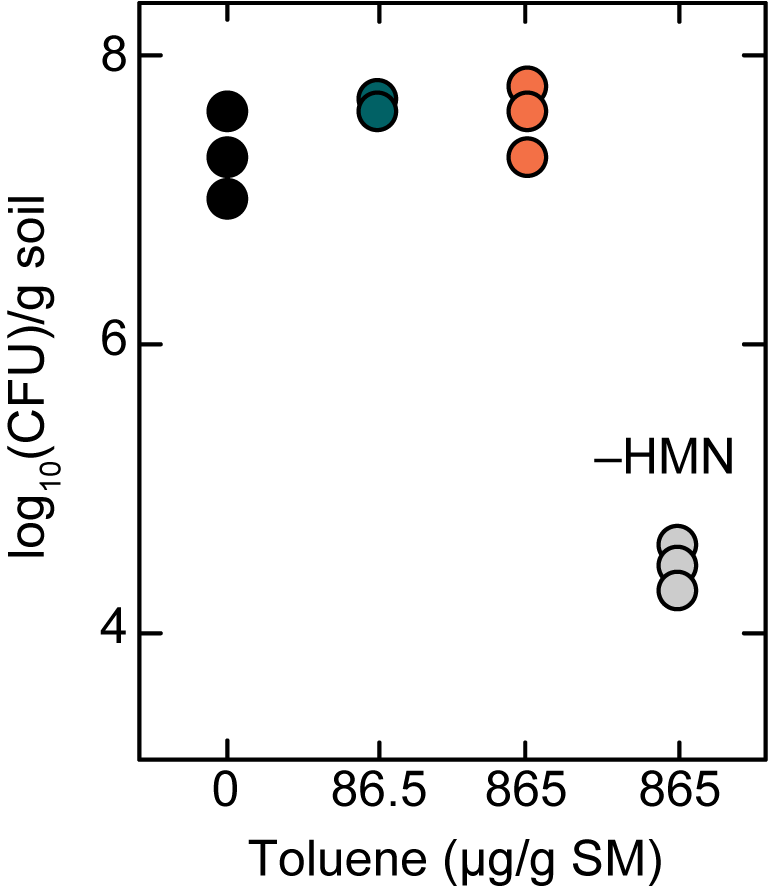


**Supplemental Figure 4**. Cell densities of SynCom 24 h after transfer to fresh soil matrix with varying toluene concentrations: 0 μg per g soil matrix (black); 86.5 μg per g SM (dark aqua); 865 μg per g SM, orange; 865 μg per g SM without 2,2’,4,4’,6,8,8’-heptamethylnonane (HMN), gray). Toluene was delivered as a solute in HMN at a concentration of 2% *v/v* in SE, except where indicated (–HMN).

**
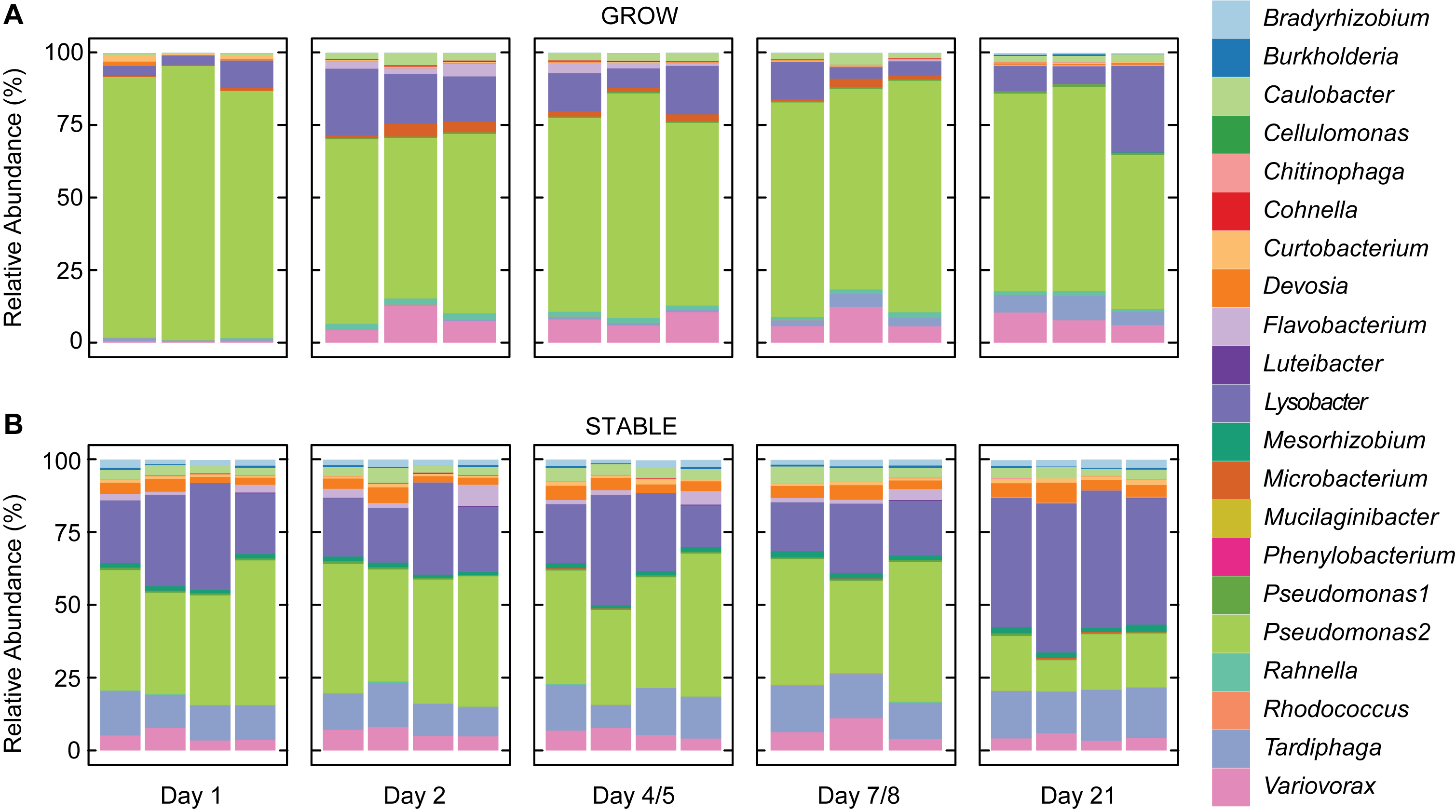
**

**Supplemental Figure 5.** Stacked relative abundances of the SynCom strains in soil microcosm in **A** grow and **B** stable phases with toluene exposure (130 μg per g soil). Grow phase was sampled on days 1, 2, 4, 7, and 21, whereas stable phase was sampled on days 1, 2, 5, 8, and 21. Columns indicate individual biological replicates. SynCom member annotations indicated on the right.

**
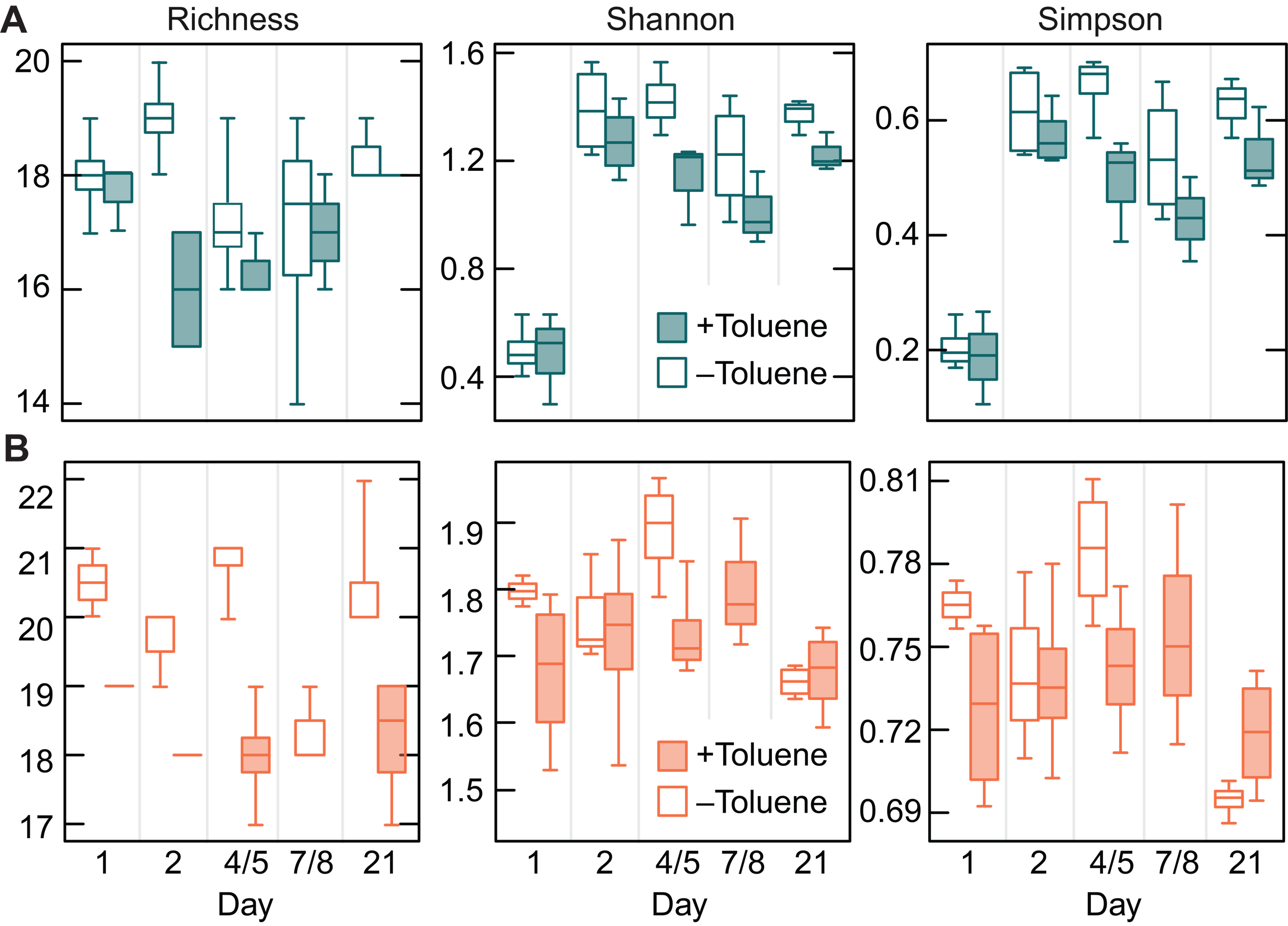
**

Supplemental Figure 6. A Bar plots showing alpha diversity of SynCom with (filled boxes) and without (empty boxes) toluene in the grow phase according to three metrics: richness (p = 0.02635, Welch’s t-test), Shannon index (p = 0.2895, Welch’s t-test), and Simpson index (p = 0.2223, Welch’s t-test). B Bar plots showing alpha diversity of SynCom with and without toluene in the stable phase according to three metrics: richness (p = 4.831$\boldsymbol{\times}$ 10^–8^, Welch’s t-test), Shannon index (p = 0.1487, Welch’s t-test), and Simpson index (p = 0.5386, Welch’s t-test).


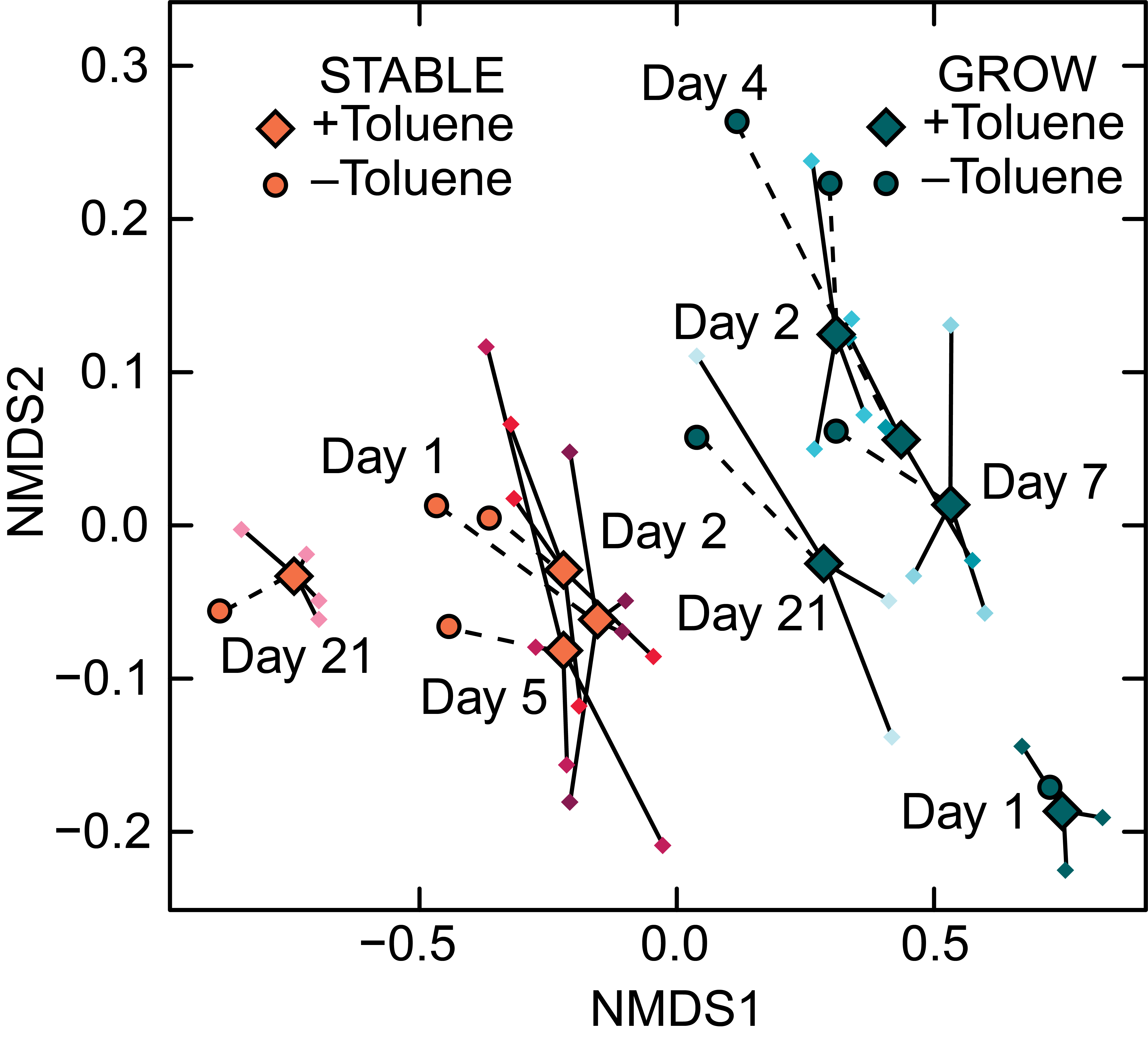


**Supplemental Figure 7**. Non-metric multidimensional scaling analysis of the SynCom species relative abundance data during the grow (dark aqua) and stable (orange) phase of growth over time in the absence (circles) and presence (diamonds) of 130 μg toluene per g soil using Bray-Curtis distance values (stress = 0.045). Data from non-toluene treated samples reproduced from the main text Figure 2. Daily centroids are shown as outlined circles or diamonds and replicates are shown as smaller circles, colored according to timepoint and community phase. Daily centroids are shown as outlined diamonds or circles, and replicates for toluene-treated SynCom are shown as smaller diamonds, colored according to timepoint and community phase.


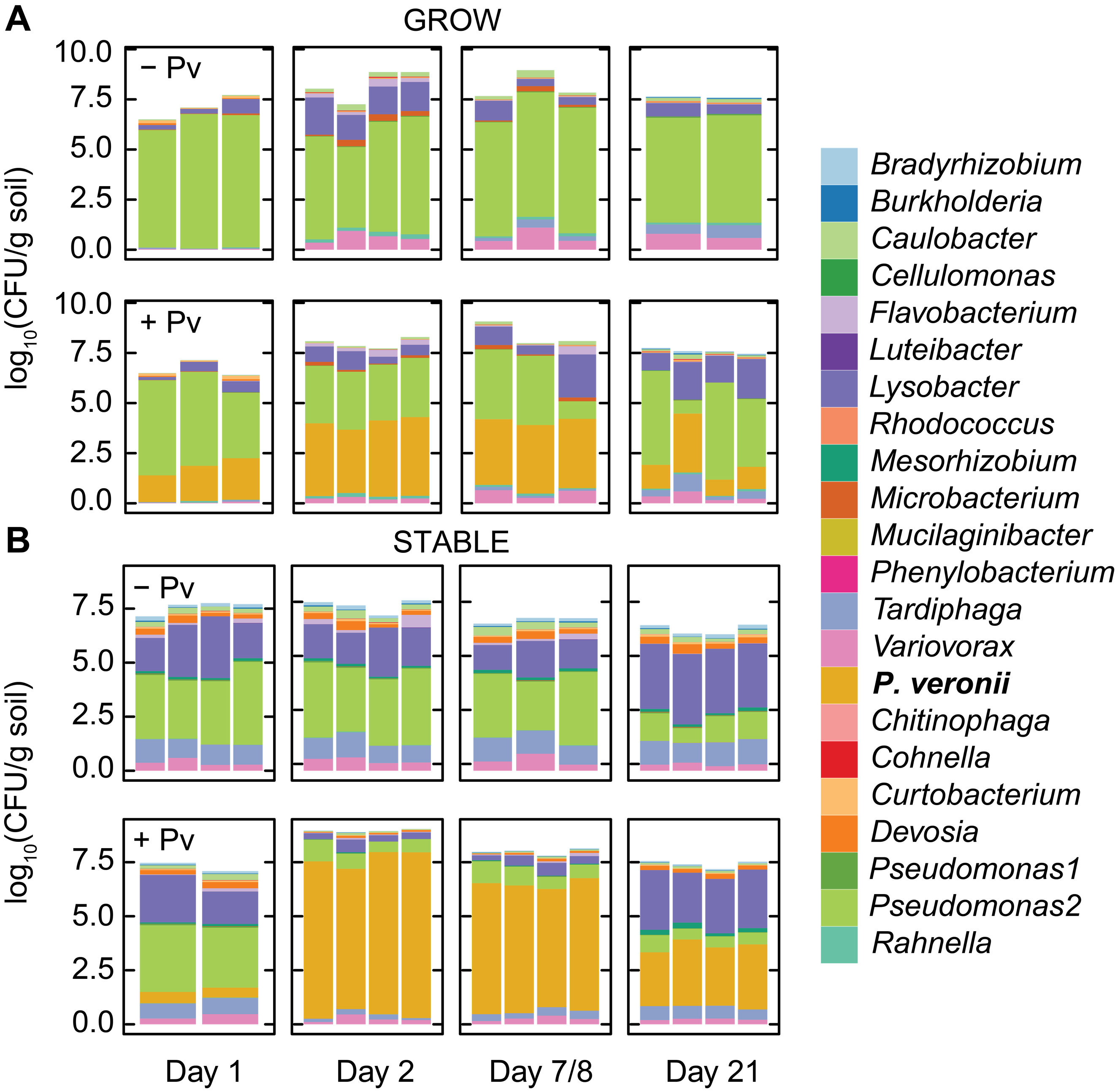


**Supplemental Figure 8**. Stacked absolute abundances of the SynCom strains in soil microcosm with toluene exposure (130 μg per g soil) in **A** grow and **B** stable phases with (+ Pv) and without (– Pv) *P. veronii* ^+^ptxD bioaugmentation. The relative abundance of each strain is depicted by the individual strain’s designated color. Grow phase was sampled on days 1, 2, 7, and 21, whereas stable phase was sampled on days 1, 2, 8, and 21. Columns indicate individual biological replicates. SynCom member annotations indicated on the right.


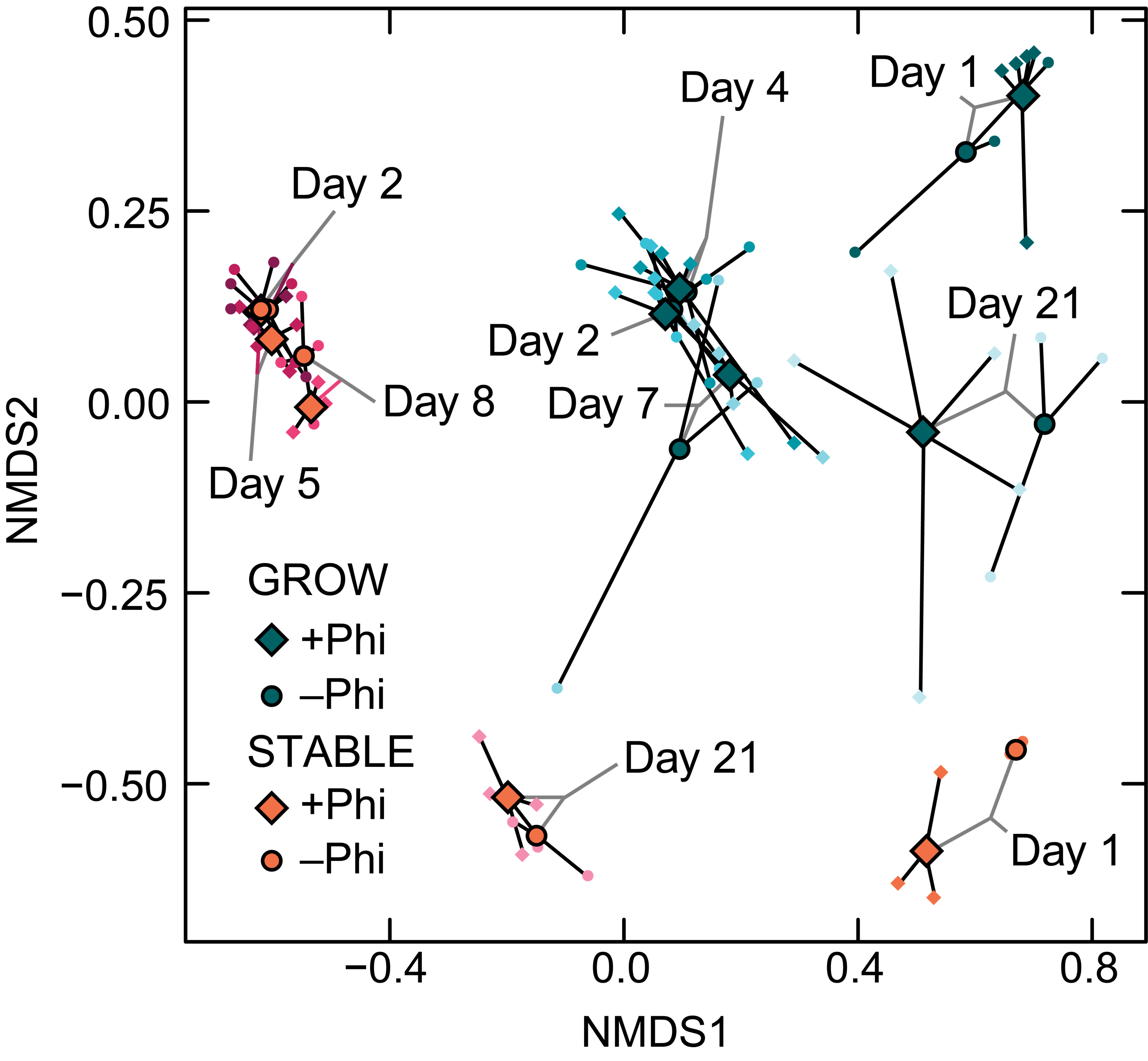


**Supplemental Figure 9**. NMDS plot based on Bray-Curtis distances (stress = 0.047) showing the composition of the toluene-remediation system in the presence (diamonds) or absence (circles) of phosphite (Phi). Community centroids are shown as outlined shapes colored according to the grow (dark aqua) and stable (orange) phase of growth.


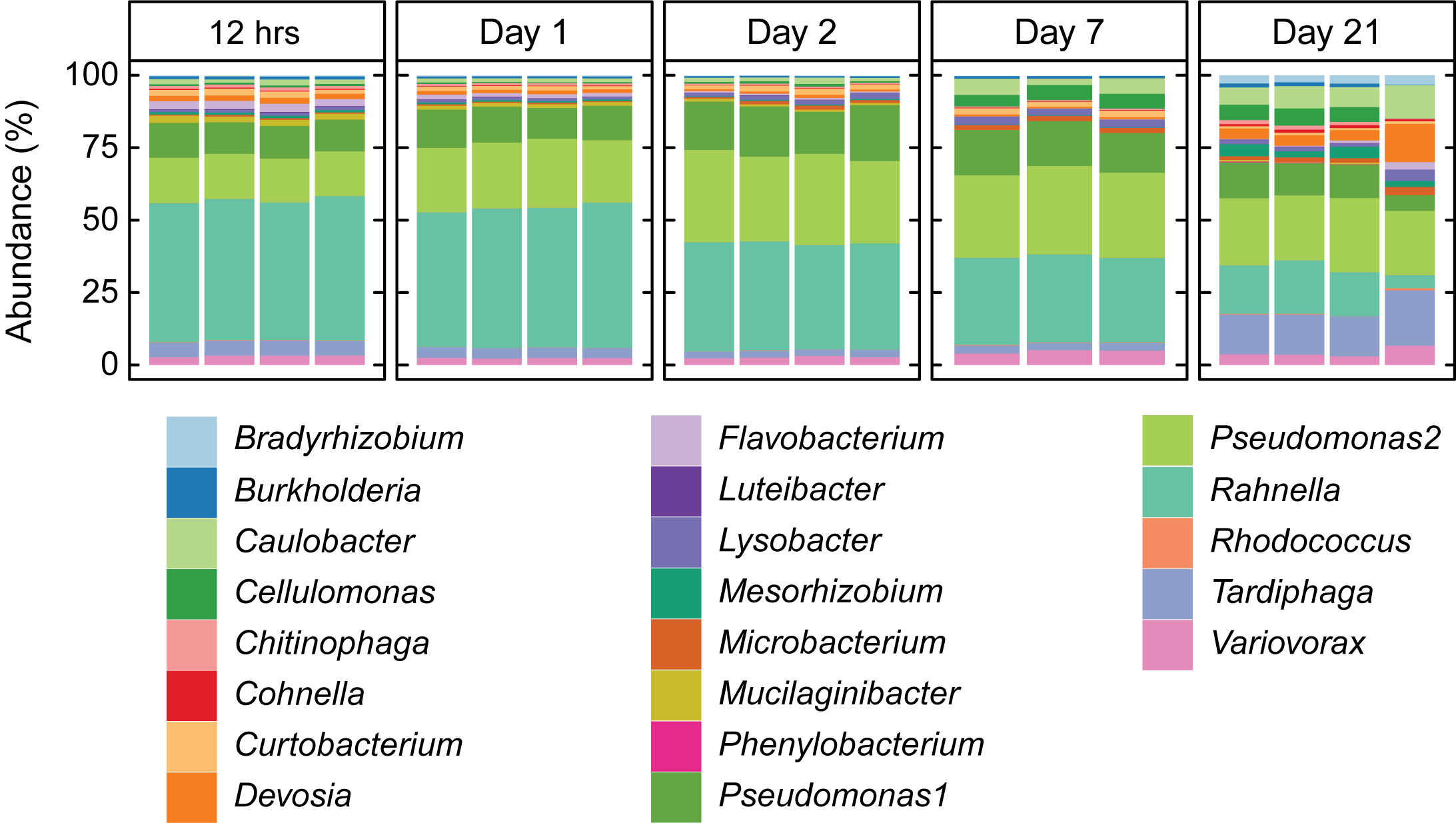


**Supplemental Figure 10**. Stacked relative abundance of SynCom members over time in soil extract (SE) medium. Columns indicate individual biological replicates. SynCom member annotations indicated on the bottom.


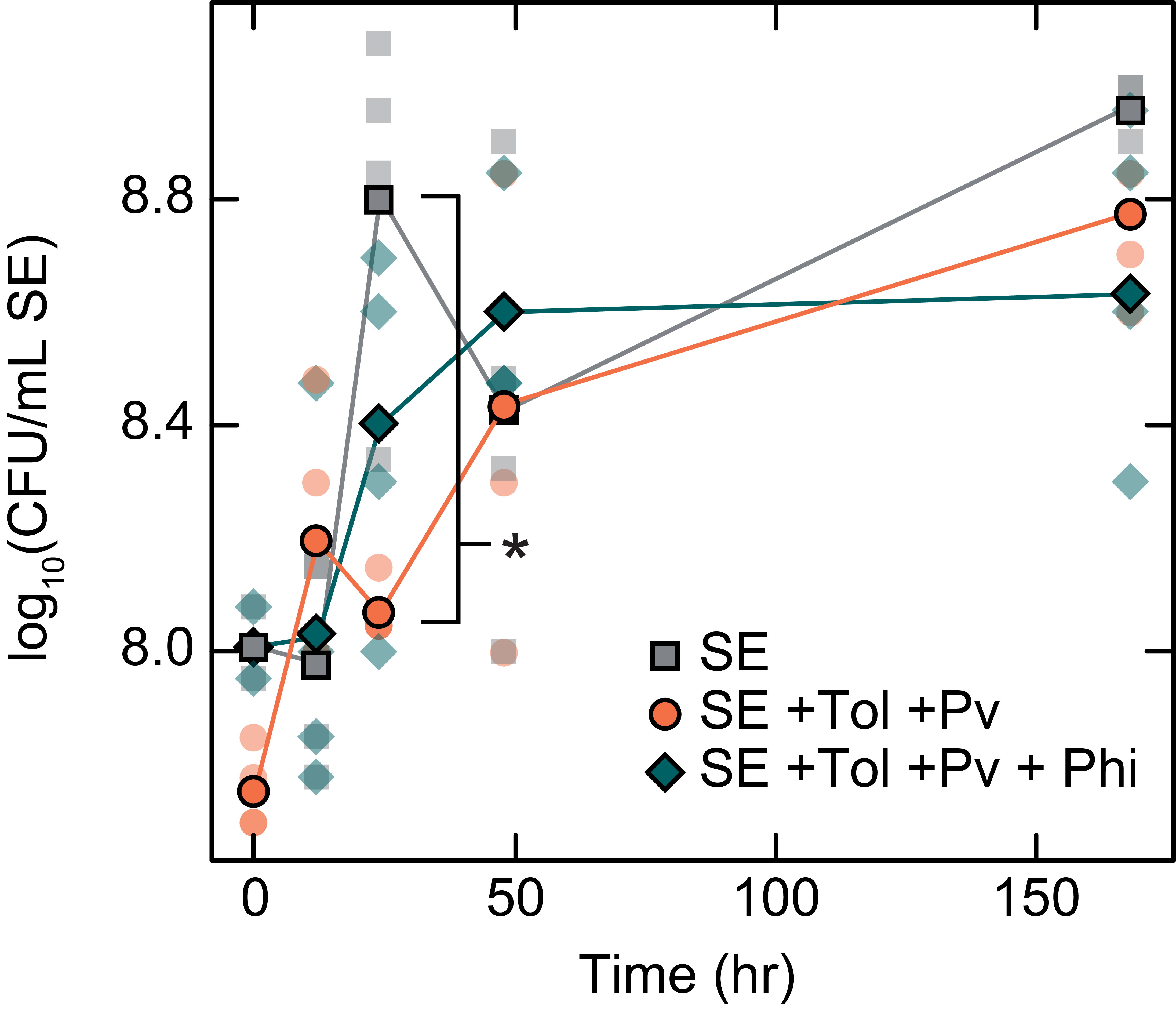


**Supplemental Figure 11**. Cell densities in soil extract (SE) determined by CFU counting over time. The SynCom community growth alone is represented with gray squares. The SynCom growth in the presence of 600 μg toluene per mL with *P. veronii ptxD^+^* in toluene-contaminated SE with (1.3 mM, orange circles) and without Phi (dark aqua diamonds) are also shown. Significant difference in cell densities between SynCom in SE and SynCom and *P. veronii* *ptxD^+^* in toluene-contaminated SE at 24 h is shown (p = 0.018, Kruskal-Wallis test).

**
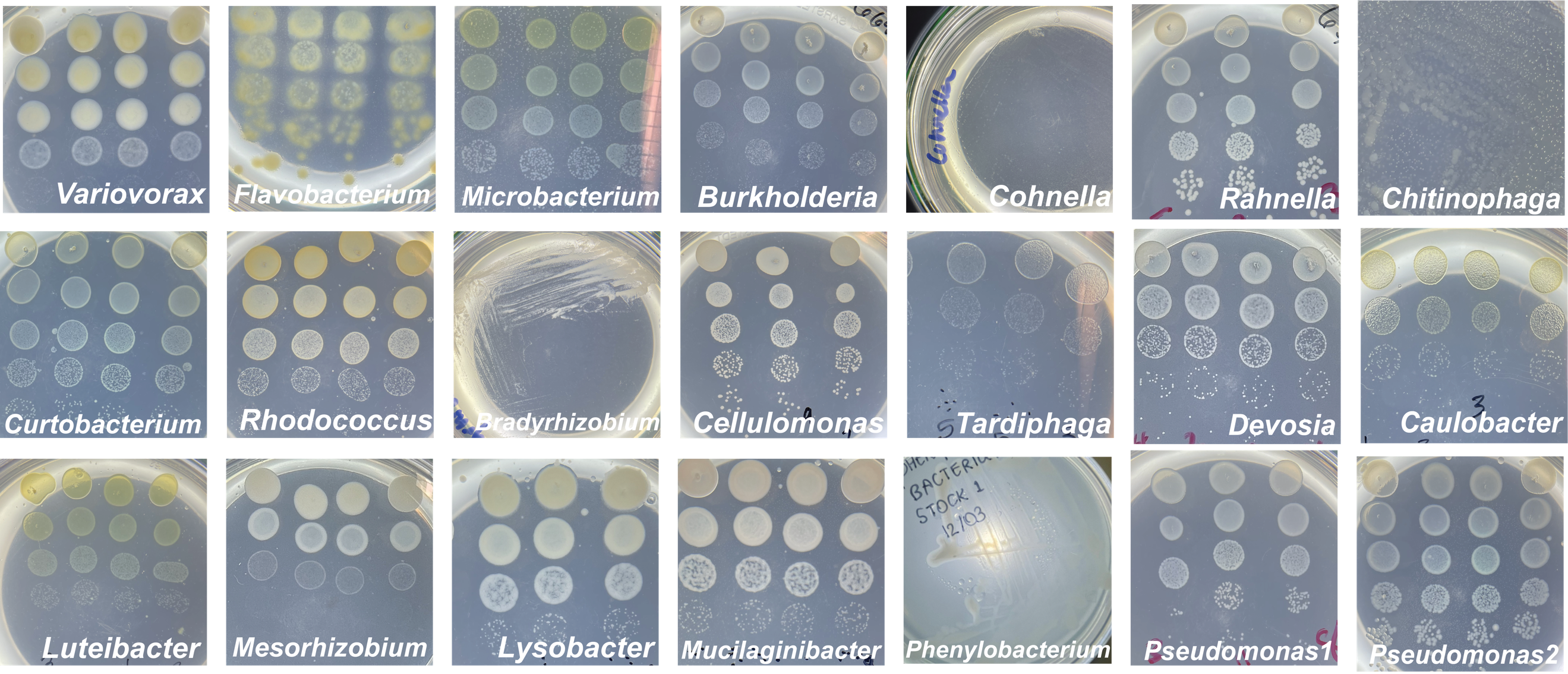
**

**Supplemental Figure 12.** The SynCom members grown on R2A agar plates.

Supplemental Table 3. pBLAST hits for *ptxD* in SynCom members’ annotated genome with E-values < 1×10^-50^.

| SynCom Member | Description | E-value | % Identity | % Query Cover |
| --- | --- | --- | --- | --- |
| *Burkholderia* | Glyoxylate/hydroxypyruvate reductase B | 4×10^-54^ | 34.81 | 93 |
| *Cohnella* | Chromosomal replication initiator protein DnaA | 9×10^-139^ | 51.61 | 76 |
| *Variovorax* | Glyoxylate/hydroxypyruvate reductase B | 4×10^-62^ | 33.23 | 97 |
|  |  |  |  |  |

**Supplemental Methods**

**Construction of *P. veronii ptxD*^+^**

*P. veronii* 1YdBTEX2 was tagged with a single copy insertion of the *ptxD* gene under control of the phosphate ABC transporter promoter. A mini-Tn*7* plasmid containing the *ptxD* gene from *P. stutzeri* WM88 was constructed using the ClonExpress II One Step Cloning Kit (Vazyme). The pUC18-Tn*7*-Gm-Amp (1) backbone was linearized by digestion with 40 units of HindIII-HF and SpeI-HF and 1 μg of DNA incubated at 37°C overnight. The resulting digestion product was isolated and concentrated using a PCR clean-up kit (Macherey-Nagel). The *ptxD* sequence of *P. stutzeri* WM88 (38) was preceded by a DNA fragment encompassing the promoter region for the phosphate ABC transporter found in *P. veronii* 1YdBTEX2 followed by a ribosome binding site. (Promoter region and *ptxD* sequence provided in **Supplemental Table 2**). The insertion was amplified by PCR (Primers provided in **Supplemental Table 2**) using Q5 Hot Start High-Fidelity DNA polymerase (New England Biolabs) and purified from the agarose gel using a gel extraction kit (Macherey-Nagel). The overlapping ends of the fragments were joined using the ClonExpress kit according to the manufacturer’s directions. After the recombination reaction was complete, the DNA product was transformed into *E. coli* DH5𝛂 cells and the resulting pUC18-Tn7-Gm-Amp-PTDH product was verified by sequencing.

The single-copy mini-Tn*7* insertion of *ptxD* into the *P. veronii* 1YdBTEX2 *glmS* gene (PVE_chr1_6133; ENA bioproject number PRJEB11417) was initiated by four-parental conjugation. Overnight cultures of *P. veronii* 1YdBTEX2 were prepared in LB (30°C), *E. coli* SM10(λpir) containing the pUX-BF13 Tn*7* helper plasmid in LB with 100 μg/mL ampicillin (37°C), *E. coli* HB101 containing the pRK2013 conjugation helper plasmid in LB with 25 μg/mL kanamycin (37°C), and *E. coli* DH5α containing the pUC18-Tn7-Gm-Amp-PTDH plasmid in LB with 100 μg/mL ampicillin (37°C). Of the 5 mL of each overnight culture, 1.5 mL was spun down at 4,025 × *g* for 4 min. The supernatant was removed and the cells were resuspended in 3 mL of fresh LB and grown at 30°C for 2 hours. After incubation, 2 mL of each of the cultures were spun down at 4,025 × g for 4 min, and the supernatant was removed and cells were resuspended in 100 μL LB. Meanwhile, 0.22 μm PVDF membrane filters (Sigma-Aldrich) were sterilized using UV light exposure for 2 minutes on each side and placed on LB plates. For conjugation, 50 μL of cells were placed on the filters in the following combinations: (1) *P. veronii* 1YdBTEX2, *E. coli* with pUX-BF13 Tn*7* helper plasmid, pRK2013 conjugation helper plasmid, and pUC18-Tn7-Gm-Amp-PTDH plasmid; (2) *P. veronii* 1YdBTEX2; and (3) and *E. coli* with pUX-BF13 Tn*7* helper plasmid, pRK2013 conjugation helper plasmid, and pUC18-Tn7-Gm-Amp-PTDH plasmid, and incubated at 30°C for 24 hours. Filters were then removed and placed in a 2 mL Eppendorf tube, to which 2 mL of sterile, 0.9% NaCl solution was added. Tubes were vortexed vigorously for 10–15 seconds and 100 μL aliquots were placed on M9 minimal media plates supplemented with 20 μg/mL gentamicin (2) plates without a carbon source. Plates were placed in an ~4 L plastic sealed container with a 2 mL toluene reservoir for vapor equilibration (toluene chamber), sealed, and grown at 30°C for 48 hours. No growth was detected from the control plates containing condition (2) nor (3). The *P. veronii* 1YdBTEX2 mutant isolates were re-streaked onto fresh M9 plates with 20 μg/mL gentamicin and toluene to isolate *P. veronii* colonies. Mutant *P. veronii* colonies were verified with colony PCR for the insertion of the mini-transposed fragment using Q5 High-Fidelity 2X Master Mix (New England Biolabs). To verify Phi oxidizing activity, 21C liquid media (3) was prepared with Phi replacing Pi as the phosphorus source (23 mM Phi) and buffer system at pH 6.8 and 10 mM succinate as the carbon source. *P. veronii* 1YdBTEX2 WT or *ptxD^+^* colonies were inoculated into 5 mL of Phi- or Pi-containing 21C media. After incubating at 30°C for 2 days, cultures were checked for growth by their culture turbidity.

**Community 16S rRNA Gene Amplicon Sequencing**

DNA was purified from thawed cell pellets using the DNeasy PowerSoil Pro kit (Qiagen) according to the manufacturer’s instructions. Purified DNA concentrations were quantified using the Qubit dsDNA BR assay kit (ThermoFischer Scientific), diluted to 10 ng/μL, and stored at -20°C. Libraries were prepared according to the Illumina 16S Metagenomic Sequencing Library protocol (4) for the V3 to V4 region of the 16S rRNA gene (Primers provided in **Supplemental Table 2**). PCR clean-up was performed using CleanNGS beads (CleanNA, The Netherlands). The gene library was indexed using Nextera XT Index Kit Set A and B (v2, Illumina Inc., USA). Libraries were supplied with 40% PhiX control, and sequenced using 300 cycle paired-end sequencing on MiSeq v3 (Illumina Inc., USA) for SM experiments and Aviti (Element Biosciences, USA) for liquid SE experiments at the Lausanne Genomic Technologies Facility.

The raw sequencing reads were assessed for quality using FastQC 0.11.9 (5), then trimmed and paired using Trimmomatic 0.39 (6) with a sliding window of 5:28. The paired reads were merged using Flash 1.2.11 (7) with a minimum and maximum overlap of 20 bp and 170 bp, respectively. To tally abundances of SynCom and inoculant strains, merged sequences were queried using the Bash “grep” command for regions of V3/V4 that were unique to each strain, as determined previously (8). Strain relative abundances were determined by first normalizing counts for each organism to the number of 16S rRNA gene operons found in the given strain then normalizing to total counts detected. Relative abundances were then combined with the CFU counting data to determine absolute abundances of the present strains.

**Toluene Quantification by GC-MS**

Toluene analysis was performed on an Agilent 8890 GC System (GC, Agilent Technologies Inc., USA) paired to an Agilent Series 5977B mass selective detector (MS). Prior to analysis, samples were removed and heated to 26°C in a dry bath for 10 minutes prior to incubation with a solid phase micro-extraction (SPME) fiber. The SPME fiber (Car-WR/PDMS, Agilent Technologies, Inc.) was conditioned prior to sampling runs by exposing the fiber to 295°C for 30 minutes (Split injection, 1:30) inside the GC inlet. For sample collection, a small gauge needle was used to puncture a hole in the 1 mL crimp vial cap and the fiber needle was inserted ~1 cm into the vial headspace and exposed for 2 minutes before injecting into the GC-MS. The gas sample was resolved on a HP-5MS Ultra inert (Agilent Technologies, Inc.) column. Acquisition parameters for GC-MS analysis were as follows: inlet temperature was 275°C and run in splitless mode; oven heated to initial temperature of 39°C and held for 4 min, then increased 15°C/min to 90°C, then increased 30°C/min to 275°C and was held for 1.43 min; transfer line temperature was 275°C. Quantification was done using MassHunter Quantitative Analysis Software (Agilent Technologies, Inc.). For the external calibration curve, 0–7.5% toluene *(v/v)* in HMN was amended to SE and SM at 2% *v*/*v*.

**Statistical Analyses**

All data analysis was performed in R (R Core Team, 2023) with packages *vegan* (9), *ggplot2* (10), and *dplyr* (11). Community composition across conditions and timepoints was analyzed using strain relative and absolute abundance data and Bray-Curtis distances determined using the *vegdist* function in *vegan* and was then ordinated using non-metric multidimensional scaling (NMDS) with function *metaMDS.* To determine differences between treatments in NMDS plots, Adonis (PERMANOVA with 999 permutations) was used with function *adonis2* in the *vegan* package. Alpha diversity was determined using *diversity* (Shannon and Simpson indices) and *specnumber* (richness) from *vegan,* and differences between conditions were evaluated using calculated p values from the Welch’s t-test*.* Enriched or depleted community members for the applied conditions were determined by inputting the relative strain abundances into a linear regression model, and outliers were identified as those with a >1 log_10_ difference in the actual values to the theoretical values determined in the model (12). All outliers identified were confirmed as outside the 99% confidence interval for the linear regression model. The effect of time and inoculant presence or absence, or time and Phi presence or absence, on toluene concentrations was evaluated using two-way ANOVA. The effect of time and Phi presence or absence on *P*. *veronii* *ptxD^+^* abundance was also assessed using two-way ANOVA.

**References**

1. Choi K-H, Gaynor JB, White KG, Lopez C, Bosio CM, Karkhoff-Schweizer RR, Schweizer HP. 2005. A Tsn7-based broad-range bacterial cloning and expression system. Nat Methods 2:443–448.

2. Sambrook J, Fritsch E, Maniatis T. 1989. Molecular cloning: a laboratory manual, 2nd ed. Cold Spring Harbor Laboratory Press.

3. Gerhardt P, Murray RGE, Costilow RN, Nester EW, Wood WA, Krieg NR, Briggs Phillips G. 1981. Manual for Methods for General Bacteriology. American Society for Microbiology, Washington, D.C.

4. Illumina. 2013. *16S metagenomic sequencing library preparation: preparing 16S ribosomal RNA gene amplicons for the Illumina MiSeq system.* Illumina, San Diego, CA.

5. Andrews S. 2010. FastQC: a quality control tool for high throughput sequence data. Babraham Bioinformatics, Babraham Institute, Cambridge, United Kingdom.

6. Bolger AM, Lohse M, Usadel B. 2014. Trimmomatic: a flexible trimmer for Illumina sequence data. Bioinformatics 30:2114–2120.

7. Magoč T, Salzberg SL. 2011. FLASH: fast length adjustment of short reads to improve genome assemblies. Bioinformatics 27:2957–2963.

8. Čaušević S, Tackmann J, Sentchilo V, von Mering C, van der Meer JR. 2022. Reproducible Propagation of Species-Rich Soil Bacterial Communities Suggests Robust Underlying Deterministic Principles of Community Formation. mSystems 7:e00160-22.

9. Dixon P. 2003. VEGAN, a package of R functions for community ecology. J Vegetation Science 14:927–930.

10. Wickham H. 2011. ggplot2. WIREs Computational Stats 3:180–185.

11. Wickham H, François R, Henry L, Müller K. 2022. dplyr: A Grammar of Data Manipulation.

12. Čaušević S, Dubey M, Morales M, Salazar G, Sentchilo V, Carraro N, Ruscheweyh H-J, Sunagawa S, Van Der Meer JR. 2024. Niche availability and competitive loss by facilitation control proliferation of bacterial strains intended for soil microbiome interventions. Nat Commun 15:2557.
